# Ultra-compact and efficient photonic waveguide bends with different configurations designed by topology optimization

**DOI:** 10.1038/s41598-024-53881-9

**Published:** 2024-03-18

**Authors:** Sabaina Irfan, Jae-Yong Kim, Hamza Kurt

**Affiliations:** School of Electrical Engineering, Korea Advanced Institute of Science and Technology (KAIST), Daejeon, 34141 South Korea

**Keywords:** Nanophotonics and plasmonics, Silicon photonics

## Abstract

Transporting light signals over the corners and sharp bends imposes high optical loss and distortion on the mode profiles. Usually, bends with larger radii are used in circuits to minimize the loss over transmission, resulting in a severe limitation in integration density. In this paper, we propose novel topology-optimized optimized L-bend and U-bend structures designed for a 220 nm silicon-on-insulator (SOI) platform. Optimized L-bends with footprints of 2.5 µm × 2.5 µm, 1.5 µm × 1.5 µm, and 1 µm × 1 µm show maximum insertion losses of only 0.07 dB, 0.26 dB, and 0.78 dB, respectively. For optimized U-bends with footprints of 3 µm × 3.6 µm, 2.5 µm × 2.5 µm, and 1.5 µm × 1.5 µm, the maximum insertion losses are 0.07 dB, 0.21 dB, and 3.16 dB. These optimized bends reduce the maximum insertion loss by over 50% compared to un-optimized arc-type bends across a broad wavelength range of 1450–1650 nm. Experimental verification of a meander line with 16 optimized U-bends (3 µm × 3.6 µm) demonstrates an averaged insertion loss of 1.23 dB in the wavelength range of 1520–1580 nm, agreeing with simulated results and indicating a high potential of loss reduction with optimized bends.

## Introduction

Over the last decades, there has been considerable growth in the field of silicon photonics technology, with advancements in devices and photonic integrated circuits (PICs) paving the way for their extensive applications such as data communication, sensing, quantum photonics^1,2^, and biomedical applications^3,4^. In particular, the silicon-on-insulator (SOI) platform has been instrumental in this progress, providing a high refractive index contrast that allows the creation of the compact design of the devices and circuits. Photonic waveguide bends, serving as fundamental components, are commonly used to guide the optical signal toward the desired pathways within these systems.

Despite the capability of high optical confinement in a silicon waveguide, the bends are still prone to significant propagation loss, particularly as smaller bend radii are employed^5^. The loss of optical mode in a bend waveguide is attributed to three main reasons (i) loss due to non-zero curvature of the waveguide (ii) mode mismatch between the input and output waveguide, and (iii) loss due to scattering and absorption in the waveguide^6^. These losses render the conventional waveguide bends incapable of being utilized in efficient and compact silicon chips. To address these challenges, several improvements over the years have been offered by extensive research in this area. The transition from sharp corners to circular bends with non-zero radius was a significant one. It was later revealed that connecting a straight waveguide to a circular bend may not be a good choice because of the mode mismatch between the straight and bend sections of the waveguide^6^. As an alternative solution, an adiabatic curvature matching approach was suggested to create a smooth match between the straight and curved waveguide sections. In addition, to further alleviate the mode mismatch, the concept of introducing a lateral offset between the input and output waveguide was also proposed^7^. Although both works successfully resolved the mode mismatch problem in the bend structure, there are still limitations to reducing the footprint for high integration. This led to explorations into non-circular bends such as Euler bends^8^, hybrid bends^9^, plasmonic bends^10^ and photonic crystals-based bends^11–13^. These bends provide non-uniform curvature for efficient light transmission, however, are not suitable for ultra-compact bends with miniaturized radii. Subwavelength gratings on waveguides and patterning with grooves are further suggested as a way to reduce the bending loss from these approaches^14^. This resulted in bends with negligible losses, including a transmission greater than 98% for photonic crystal-based L-bends with almost zero radius of curvature^11^. However, increased fabrication steps, cost, and radius of curvature are inevitable with these methods. With the evolution in the field of meta-materials, nearly perfect bending waveguides with insertion loss less than 0.2 dB are also realized with anisotropic epsilon-near-zero metamaterials^15^. Photonic crystal-based right-angled wire waveguide bends are suggested to dramatically reduce the bending loss from curved waveguide bends and to increase the integration density on chips^16,17^. Due to the fabrication complexities required for the above-mentioned methods, an optimal bend designed with an analytical approach is favorable.

Inverse design and other intelligent algorithms are now reshaping the landscape of devices available to nano-photonics. Gradient-based inverse design algorithm is used to automatically design multifunctional devices such as grating couplers^18^, polarization rotator^19^, resonators and cavities^20^, photonic switches, and devices based on mode and wavelength division multiplexing^21,22^. Inverse design approaches are also being utilized for the design of waveguide bends. Direct binary search algorithm^23^, direct range search algorithm^24^, objective first algorithm^25^, and topology optimization^26^ are used to make efficient bends with reduced footprint. By exploring the studies on waveguide bends we found that much of the existing literature appears to focus on multi-mode waveguides and the objective was mostly to reduce the insertion loss and intermodal coupling^24–29^. While the majority of research has contributed to multi-mode waveguide bending, the advancement of bend designs for single-mode propagation using inverse design methods remains relatively under-explored. Some studies also explore reducing insertion loss of single-mode waveguide bends by combining with variable adiabatic semi-natural spline shapes, however, this resulted in a size penalty, and the bend radii were limited to 2 μm^30^. To the best of our knowledge, no study reports working on ultra-compact single-mode waveguide bends with radii less than 2 μm with inverse design.

In this paper, we propose a topology optimization-based approach for designing optimal single-mode waveguide bends in a 220 nm silicon-on-insulator (SOI) platform with ultra-compact sizes. The ultimate aim of this work is to minimize the propagation loss along the bending or corner regions (including mode-mismatch, radiation, and sidewall roughness). The model defines the mode coupling between the input and output waveguide as the figure of merit and tries to maximize this coupling. We subsequently show that the resulting structure is an optimal bend with very low propagation and insertion loss over a wide bandwidth. Comparison of the insertion loss of the optimal bends with bends designed using various inverse design approaches, including 1 dB for direct binary search (R = 1 µm, multimode)^23^, 0.04 dB for direct range search (R = 9.35 µm, multimode)^24^, and 0.023 dB for topology optimization (R = 5.5 µm, multimode)^26^, demonstrate the superior performance of our topology-optimized nano-photonic bends (R = 2.5 µm, single mode). The measured average experimental transmission of a meander line with 16 optimized bends shows an insertion loss of 1.24 dB in wavelength range of 1450-1650 nm. The simulated value shows maximum loss to be 1.23 dB in the wavelength range of 1450–1650 nm therefore consistent data is obtained experimentally.

## Methods

**Figure 1:**
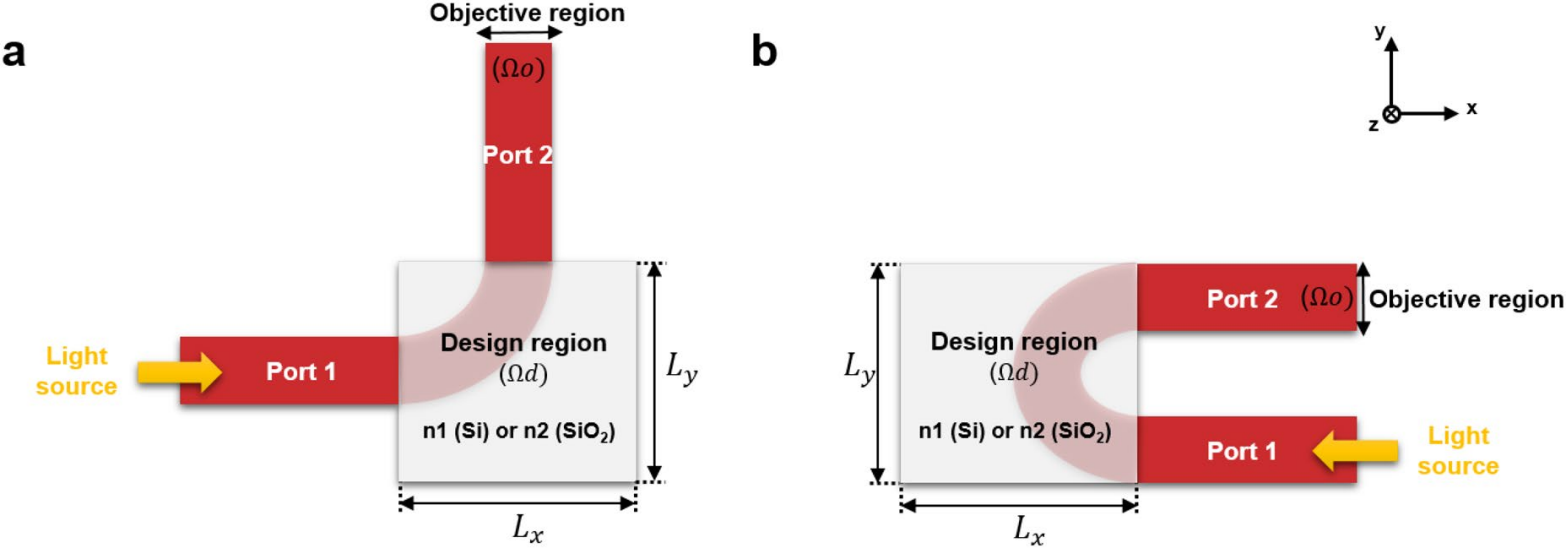
(**a**) Schematic design of L-bend and (**b**) U-bend.

The design model for waveguide bends is shown in Fig. 1. The structural parameters are the following: n_1_ = 3.47, n_2_ = 1.44, and variable design region lengths L_x_ and L_y_. The device consists of an input and output waveguide with a width of 500 nm. The source is transverse electric (TE)-polarized with a bandwidth of 200 nm and a center wavelength of 1550 nm.

Among the various available inverse-designed methodologies such as objective-first algorithm, particle-swarm optimization, and parametric optimization, topology optimization was chosen because of the unparalleled design freedom it offers. Originating from the field of solid mechanics, this method now has attracted considerable attention in many areas of physics including the photonics field^31^. This powerful technique reshapes the material layout within a device's design space to achieve optimal performance, often leading to creative and unexpected topologies. Incorporating the electromagnetic Maxwell solver in the finite-difference time-domain (FDTD), topology optimization operates alongside gradient-based optimization tools. It generates different nanophotonic structures as candidate solutions to meet a predefined figure-of-merit (FOM). By just specifying the optimization area, topology optimization refines the material distribution on a point-by-point basis, aiming to design highly efficient structures. Among various inverse design algorithms, topology optimization stands out for several reasons. The direct binary search (DBS) approach is limited by a pixel-by-pixel alteration mechanism, and particle swarm optimization introduces block constraints. On the other hand, topology optimization overcomes these limitations by applying filters concurrently during the optimization process. This not only saves computational time but also allows for the design of broadband devices, overcoming the single-wavelength operation limitation of objective-first algorithms^32^.

Despite these advantages, owing to the non-intuitive nature of the generated structures with topology optimization, limitations in the structure design are introduced for the design to conform to the fabrication constraints. Any structural design problem using topology optimization is represented as a continuous constrained optimization problem^31,33^, which is written as follow

1 $$\underset{\xi }{{\text{max}}}\Phi \left(\upxi ({\text{r}}) \right), \Phi : {\left[\mathrm{0,1}\right]}^{\Omega d}\to R$$

$$s.t. {c}_{i}\left(\xi \right)= 0, {c}_{i} : {[\mathrm{0,1}]}^{\Omega d} \to R, i \in \{0, 1, ..., {N}_{i}\}, {N}_{i}\in {N}_{0}$$

$${c}_{j} (\xi ) < 0, {c}_{j} : {[\mathrm{0,1}]}^{\Omega d}\to R, j \in \{0, 1, ..., {N}_{i}\}, {N}_{j} \in {N}_{0}$$

Here ξ(r) ∈ [0, 1] denotes a continuous design field within the design domain $(\Omega d)$, over which the function Φ, called the Figure of Merit (FOM), is maximized. The equations ${c}_{i}=0$ denote ${N}_{i}$ equality constraints, and ${c}_{j}=0$ denote ${N}_{j}$ inequality constraints. The equality constraints represent certain conditions such as feature size limitations that should be satisfied in the optimization process. The inequality constraints represent restrictions on design space. Equation (1) suggests that for a given problem, one must select a FOM, Φ(ξ), which provides a reliable measure of the performance of the design. Further, one must select a set of functions, ${c}_{i} (\xi )$ and ${c}_{j} (\xi )$, providing reliable measures of all constrains associated with the design problem.

In our inverse design methodology, the Figure of Merit (FOM) is defined using a transmission field monitor, specifically located within the objective domain $\Omega$ o. This monitor assesses the mode overlap to the fundamental TE mode of the waveguides and tries to maximize its transmission. FOM is a function of electric field (E), magnetic field (H), and device permittivity ${\varepsilon }_{r}$ defined based on Poynting vectors, representing the electromagnetic (EM) field

2 $${\mathrm{S }({\text{E}},\mathrm{ H}) ={\text{E}}\times {\text{H}}}_{{\text{T}}}+{{\text{E}}}_{{\text{T}}}\times {\text{H}}$$

The objective function Φ(E, H) is then defined as the magnitude of the Poynting vector:

3 $$\Phi \, \left( {{\text{E}},{\text{ H}}} \right) \, = \, \left| {{\text{S }}\left( {{\text{E}},{\text{ H}}} \right)} \right|$$

To ensure that the actual EM fields (E and H) are aligned with the fundamental TE mode, the objective is to maximize Φ at each position xo within $\Omega$ o. Finally, the total Figure of Merit (FOM) is defined as the integral of the objective function over the entire objective domain

4 $$\Phi = \int\limits_{\Omega 0} {\Phi (E({\text{x}}o)),(H({\text{x}}o))d^{3} {\text{x}}o}$$

The optimization problem is subjected to Maxwell wave equation

5 $$\nabla x\nabla xE-{{K}_{O}}^{2}{\varepsilon }_{r}E=-j\omega {\mu }_{o}J$$

as a constraint which ensures physical validity of designed structure. To solve optimization problem in the form of Eq. (1) a continuous design field, ξ(r) is used to control the material distribution in Ω through the interpolation function,

6 $${\varepsilon }_{r}(\xi (r))) = {\varepsilon }_{r,Si{O}_{2}}+ \xi (r) ({\varepsilon }_{r, Si} - {\varepsilon }_{r, Si{O}_{2}})$$

where ${\varepsilon }_{r, Si}$ and ${\varepsilon }_{r, Si{O}_{2}}$ denote the relative permittivity of silicon and silicon dioxide, respectively. The above equation defines two equality constraints i.e., ξ = 0 ⇔ ${\varepsilon }_{r}$ = ${\varepsilon }_{r, Si{O}_{2}}$ and ξ = 1 ⇔ ${\varepsilon }_{r}$ = ${\varepsilon }_{r, Si}$ and an inequality constraint ξ = 0 to our optimization problem.

To address the continuously constrained optimization problem described above, topology optimization uses an adjoint-based sensitivity analysis, which requires only solving a single (adjoint) equation for the FOM and one for each constraint in the optimization problem to maximize the desired FOM. Such algorithms require knowledge of the gradients (sensitivities) of Φ, *ci*, and *cj* with respect to the design field ξ. These sensitivities can also be approximated using finite differences, which entails solving the system equations for perturbations of each of the design variables for each design iteration. However, doing so is in most cases is time consuming due to the large number of equations that must be solved. In our optimizations, the inverse design is carried out using the Lumopt classes provided by the software package Lumerical. A base file provides the input/output geometry and define the optimization volume. Each FDTD mesh cell in the discretized optimization volume becomes a parameter. These parameters are used by optimization class. It takes the defined figure of merit and perform optimization based on wavelength and propagation characteristics defined in the set-up classes. A heavy-side filter is employed in optimization class to smooth the sharp edges and corners in our design, with a specified filter radius of 100 nm. It is also possible to define minimum feature size constraint in our optimization process by adding a penalty term to figure of merit. The minimum feature size is specified to be 150 nm for the resulting structure to be easily fabricated with the e-beam lithography process. The resulting structure’s performance strongly depends on the initial condition used for designing the structure. It is possible to fill the design area with the average material parameters of the two materials used in the design. In our case, we give the bending structure as the initial guess for the optimization to start.

## Results

Different structures for L-bends and U-bends were generated by varying the design area and the design regions were varied from 3 μm × 3.6 μm to 1 μm × 1 μm to realize ultra-compact bends. These initial structures were firstly optimized in two-dimensional (2D), which took about 170 iterations and 6–7 h to converge. The generated bending structures with topology optimization were tested for their transmission and insertion loss with FDTD. The optimized structures in 2D were given as initial guesses for three-dimensional (3D) optimization to start and it took about 70 iterations and 10 to 12 h to complete on average. The introduction of penalty function in FOM to ensure adherence to fabrication constraints increased the simulation times.

### Designed structures

#### L-bends

**Figure 2:**
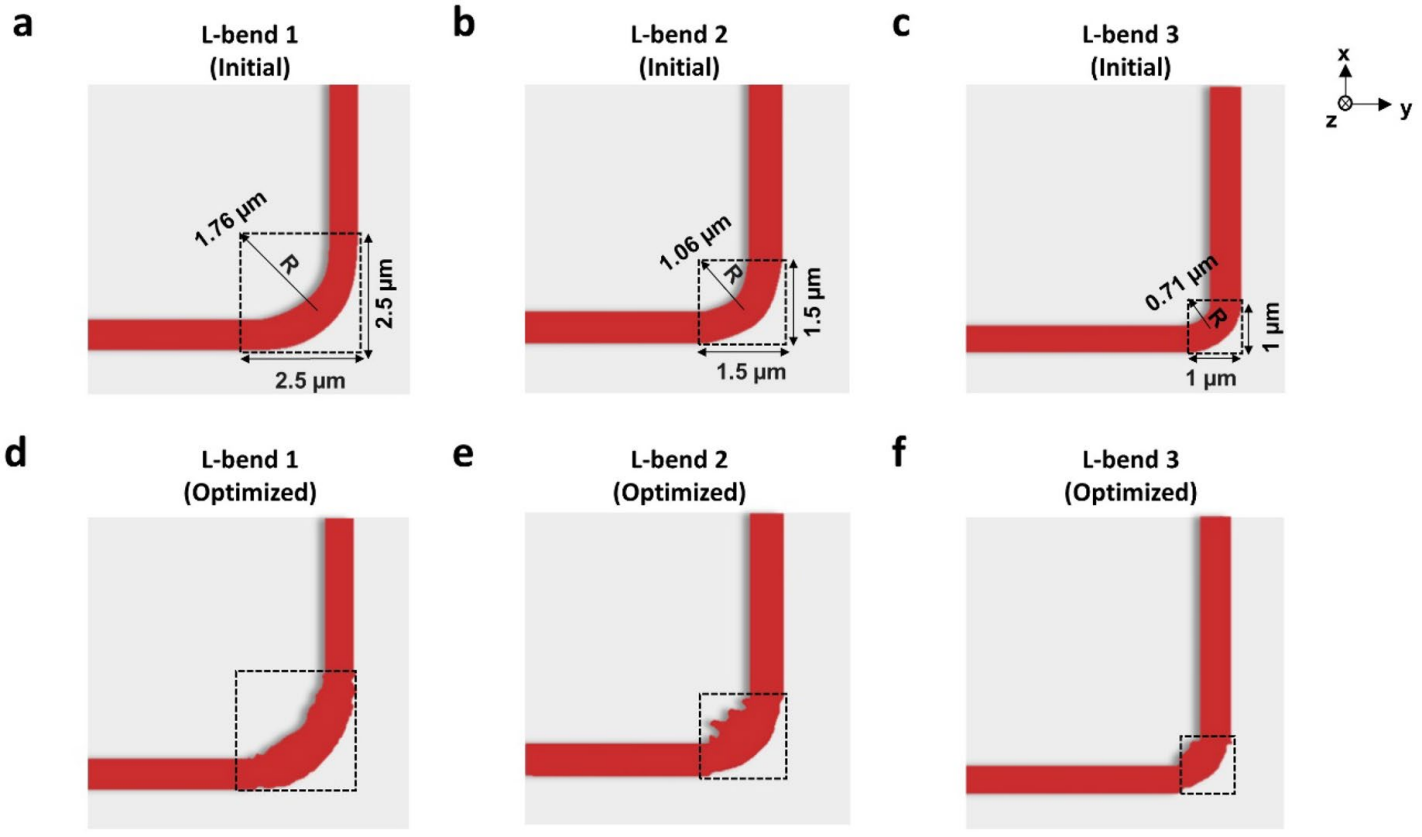
Initial structures of (**a**) L-bend 1 (radii: 1.76 µm), (**b**) L-bend 2 (radii: 1.06 µm), (**c**) L-bend 3 (radii: 0.71 µm). Topology-optimized structures of (**d**) L-bend 1 (radii: 1.76 µm), (**e**) L-bend 2 (radii: 1.06 µm), and (**f**) L-bend 3 (radii: 0.71 µm).

In Fig. 1, L_x_ and L_y_ were varied to make different radii L-bends, and an arc bend of equivalent bending radius was given as an initial guess for optimization. Three different footprints for L-bends are made. These include L_x_ = 2.5 µm and L_y_ = 2.5 µm, L_x_ = 1.5 µm and L_y_ = 1.5 µm, and L_x_ = 1 µm and L_y_ = 1 µm. In subsequent definitions, we call them L-bend 1, L-bend 2, and L-bend 3 respectively. The radii of bends are measured as half of the diagonal of squared design region. The initial and optimized structures are shown in Fig. 2. The optimization works to modify this structure to maximize the coupling and minimize the mode mismatch between the input and output waveguide for a broad wavelength range of 1450–1650 nm.

**Figure 3:**
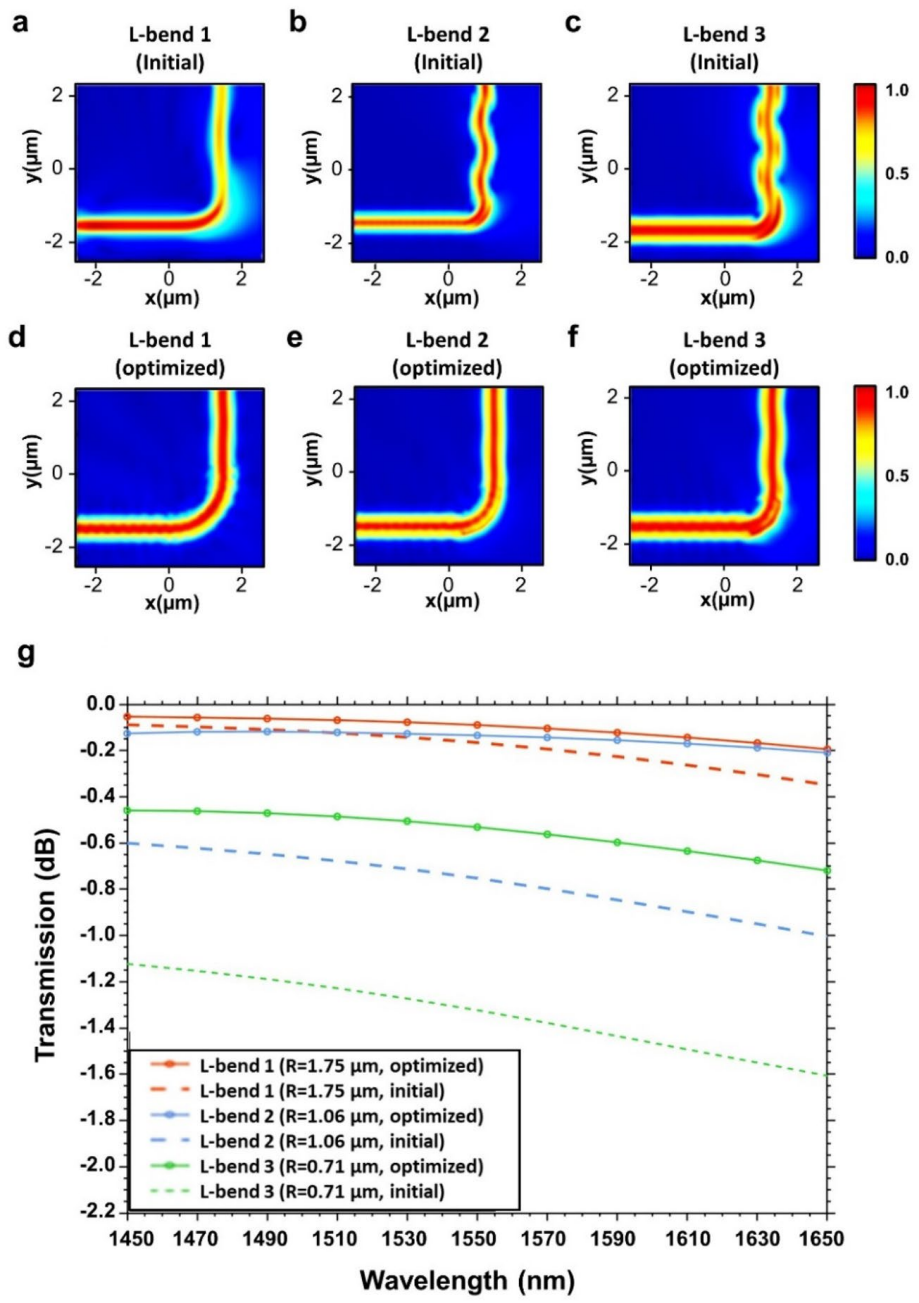
Simulated electric field profiles of (**a**) initial L-bend 1 (radii: 1.75 µm), (**b**) initial L-bend 2 (radii: 1.06 µm), (**c**) initial L-bend 3 (radii: 0.71 µm). (**d**) topology-optimized L-bend 1 (radii: 1.75 µm), (**e**) topology-optimized L-bend 2 (radii: 1.06 µm), and (**f**) topology-optimized L-bend 3 (radii: 0.71 µm). (**g**) Comparison of simulated insertion losses of optimized L-bends and un-optimized L-bends with varied radii.

The simulated electric field profiles for the conventional L-bends and optimized L-bends (the initial guess for 2D optimization) are shown in Fig. 3a–f. It suggests that as the bending radii of the L-bend decreased, the loss of un-optimized arc-type bends increased and the mode profile was distorted. The topology optimization works to decrease the mode distortion while simultaneously increasing the transmission. By looking at the generated design we can see the algorithm generates a mismatch between the curve and straight section of the waveguide to preserve the mode profile which has long been suggested as a way to decrease the mode mismatch in different optical bends^6^.

The performances of L-bend structures were rigorously assessed through transmission analysis, both in their initial states and optimized states which are described in Fig. 3g. The trend highlights a significant improvement in insertion loss with the implementation of topology-optimized bends compared to un-optimized arc-bends. For bending radii of 1.75 µm, 1.06 µm, and 0.71 µm, the optimized L-bends exhibit a percentage decrease in maximum insertion loss of 81.2%, 64.8%, and 51.55% respectively in the simulation results. For the L-bend 1, the un-optimized configuration exhibited a maximum transmission of 97.9% and a minimum transmission of 92.2%. After optimization, these values significantly increased, with a maximum transmission of 98.7% and a minimum transmission of 95.6%, showcasing the optimization's ability to enhance both the best-case and worst-case scenarios. Similarly, L-bend 2, in its initial form, displayed a maximum transmission of 87.01% and a minimum transmission of 79.1%. Following optimization, these values improved substantially to 97.1% and 95.2% respectively. The L-bend 3, when un-optimized, achieved a maximum transmission of 77.2% and a minimum transmission of 68.9%. Post-optimization, these values rose notably to 89.1% and 83.5%, underlining the optimization's capacity to enhance performance, especially in scenarios where transmission rates were previously comparatively low.

#### U-bends

**Figure 4:**
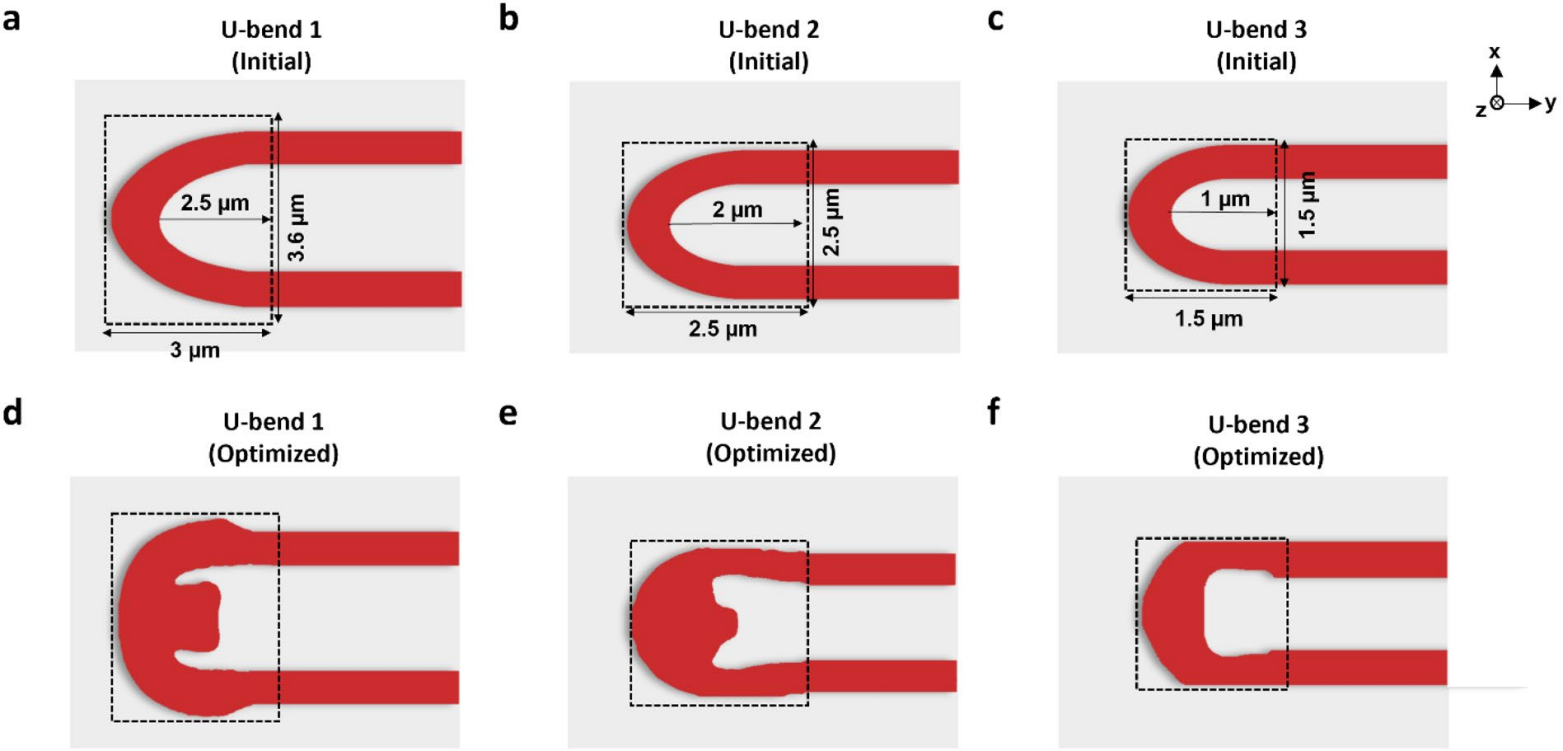
Initial structures of (**a**) U-bend 1 (radii: 2.5 µm), (**b**) U-bend 2 (radii: 2.0 µm), and (**c**) U-bend 3 (radii: 1.0 µm). Topology-optimized structures of (**d**) U-bend 1 (radii: 2.5 µm), (**e**) U-bend 2 (radii: 2.0 µm), and (**f**) U-bend 3 (radii: 1.0 µm).

Based on the improvement observed in bending light by 90° with optimized structures, another configuration for input and output waveguide as shown in Fig. 1b was specified to make a U-bend capable of bending light by 180 degrees. Different optimized structures were generated by varying the design region for the U-bends. The design region was varied from 3.0 µm × 3.6 μm to 1.0 µm × 1.0 μm in order to realize ultra-compact bends. These bends are called U-bend 1, U-bend 2, and U-bend 3 in later parts of the paper. The outer radius is measured from the arc waveguide termination point to the end of design region and the optimized configurations in similar footprints are assumed to have similar radii. The initial arc-bends and the resulting topology-optimized structures are shown in Fig. 4a–f.

**Figure 5:**
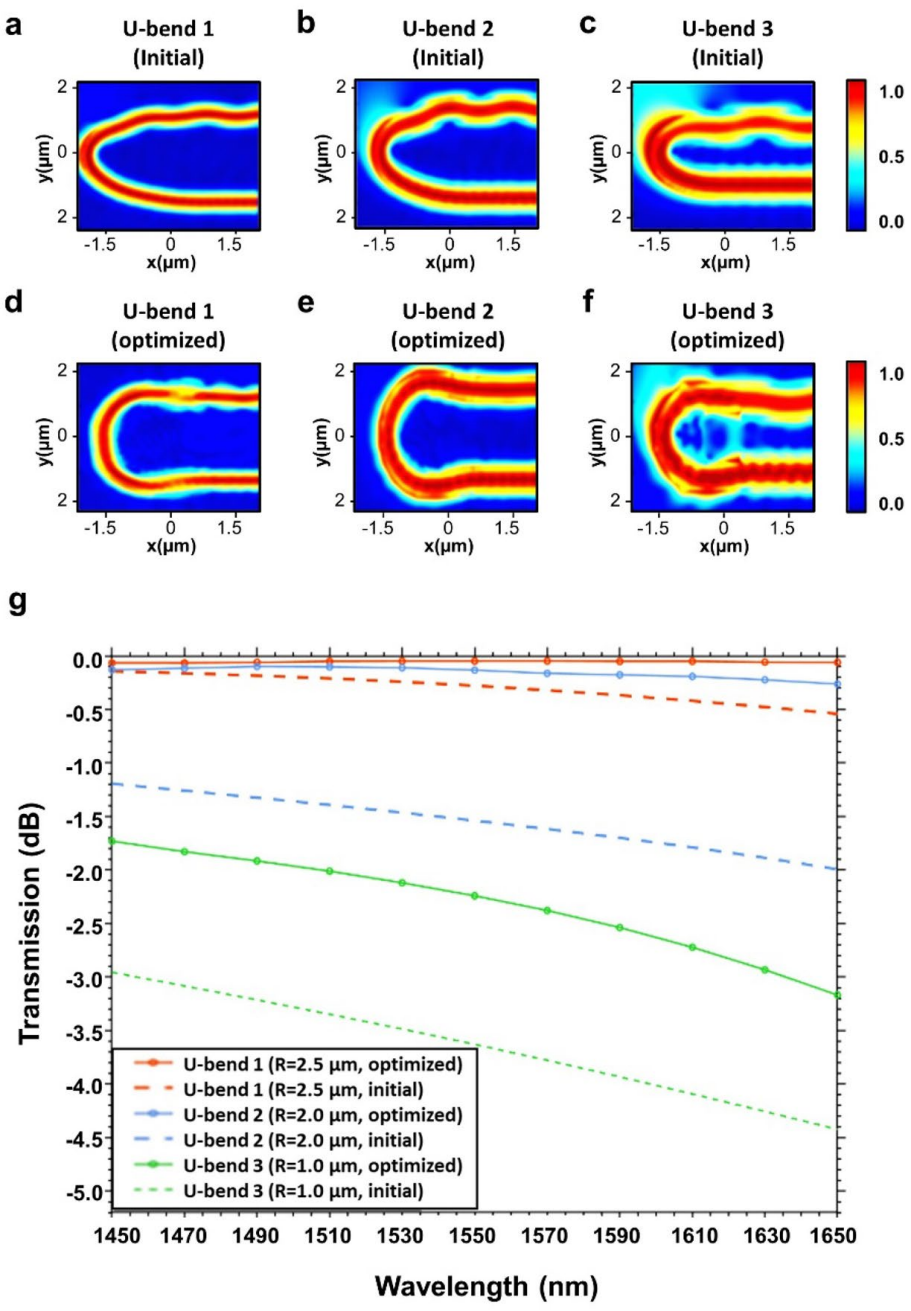
Simulated electric field profiles for (**a**) initial U-bend 1 (radii: 2.5 µm), (**b**) initial U-bend 2 (radii: 2.0 µm), (**c**) initial U-bend 3 (radii: 1.0 µm), (**d**) topology-optimized U-bend 1 (radii: 2.5 µm), (**e**) topology-optimized U-bend 2 (radii: 2.0 µm), and (**f**) topology-optimized U-bend 3 (radii: 1.0 µm). (**g**) Comparison of simulated insertion losses of optimized and un-optimized U-bends with varied radii.

The simulated electric field profiles for the conventional U-bends and the topology-optimized U-bends with different radii are shown in Fig. 5a–f. Figure 5a–c shows arc-type U-bends which are also the initial structure for our optimization process. It can be seen that there is high transmission loss and mode distortion at output. The reason for mode profile distortion is that, in single-mode waveguides, presence of modes beyond the fundamental mode, possibly including radiation modes or other higher-order guided modes can be excited due to mode coupling caused by the bend's curvature. Secondly, in a small bending radius, the waveguide cross-sectional shape can deviate from its ideal rectangular shape. This geometric distortion affects the waveguide's refractive index profile, which in turn alters the propagation characteristics of the guided mode. One other reason is that when the light propagates through the bend, the rays traveling along the inner and outer sides of the bend experience different path lengths. This discrepancy leads to phase variations between different portions of the mode profile, resulting in interference effects and mode profile distortion. The equivalent topology-optimized U-bends retain the mode profile and have a high transmission at output.

The comparison of transmission for U-bends in their optimized and un-optimized configuration is shown in Fig. 5g. The performance enhancement achieved through topology optimization is strikingly evident in the transmission analysis of U-bend structures. With bending radii of 2.5 µm, 2 µm, and 1 µm, the optimized U-bends achieve percentage reductions in maximum insertion loss of 84.89%, 73.8%, and 29.9% respectively. In its un-optimized form, the U-bend 1 exhibited a maximum transmission of 96.7% and a minimum transmission of 88.2%. Following the optimization process, these values experienced significant escalation, reaching a maximum transmission of 98.7% and a minimum transmission of 98.5%. Notably, in the reduced design region of U-bend 2, the average transmission of the optimized structure slightly decreased to about 95.5%. Even with this reduction, the optimized U-bend significantly outperformed its un-optimized counterpart, showcasing a maximum transmission of 97% and a minimum transmission of 94.1%. The un-optimized U-bend 2 only reaches a maximum transmission of 76.3% and a minimum transmission of 62.7%. In the most challenging scenario, where the design region was further decreased to 1.5 μm × 1.5 μm (U-bend 3), the un-optimized U-bend had a maximum transmission of 51.1% and a minimum transmission of 35.7%. However, after the topology optimization, these figures improved significantly to 67.1% and 48.2%, respectively. Despite the inherent challenges posed by reduced design areas and tighter bending radii leading to increased bending losses, the optimized U-bend designs showcased substantial advancements compared to their un-optimized counterparts. This comparison signifies the effectiveness of topology optimization in enhancing signal integrity and preserving the quality of transmitted signals, especially in bends with smaller radii. Figure 5g also shows that as the wavelength increases, the insertion loss for the bends increases. This increase can be attributed to several physical phenomena such as wavelength-dependent absorption in dielectric materials, increased radiative loss with an increase in wavelength, and the existence of higher-order modes in waveguides for larger wavelengths. It's crucial to acknowledge that our optimization encompasses a wide spectrum, which introduces subtle variations across wavelengths while enhancing performance. Hence, achieving a perfectly flat curve for insertion loss across all wavelengths presents a unique challenge. The optimization process in our case is tailored for a broad wavelength range within a confined space. The algorithm aims to devise a structure that minimizes insertion loss across this extensive range. This complexity might be the reason for the absence of a completely flat curve for optimized bends. Despite not achieving a perfectly uniform curve, the optimized bends still exhibit significantly lower insertion loss compared to their un-optimized counterparts.

### Meander lines utilizing the optimized U-bends with experimental verification

**Figure 6:**
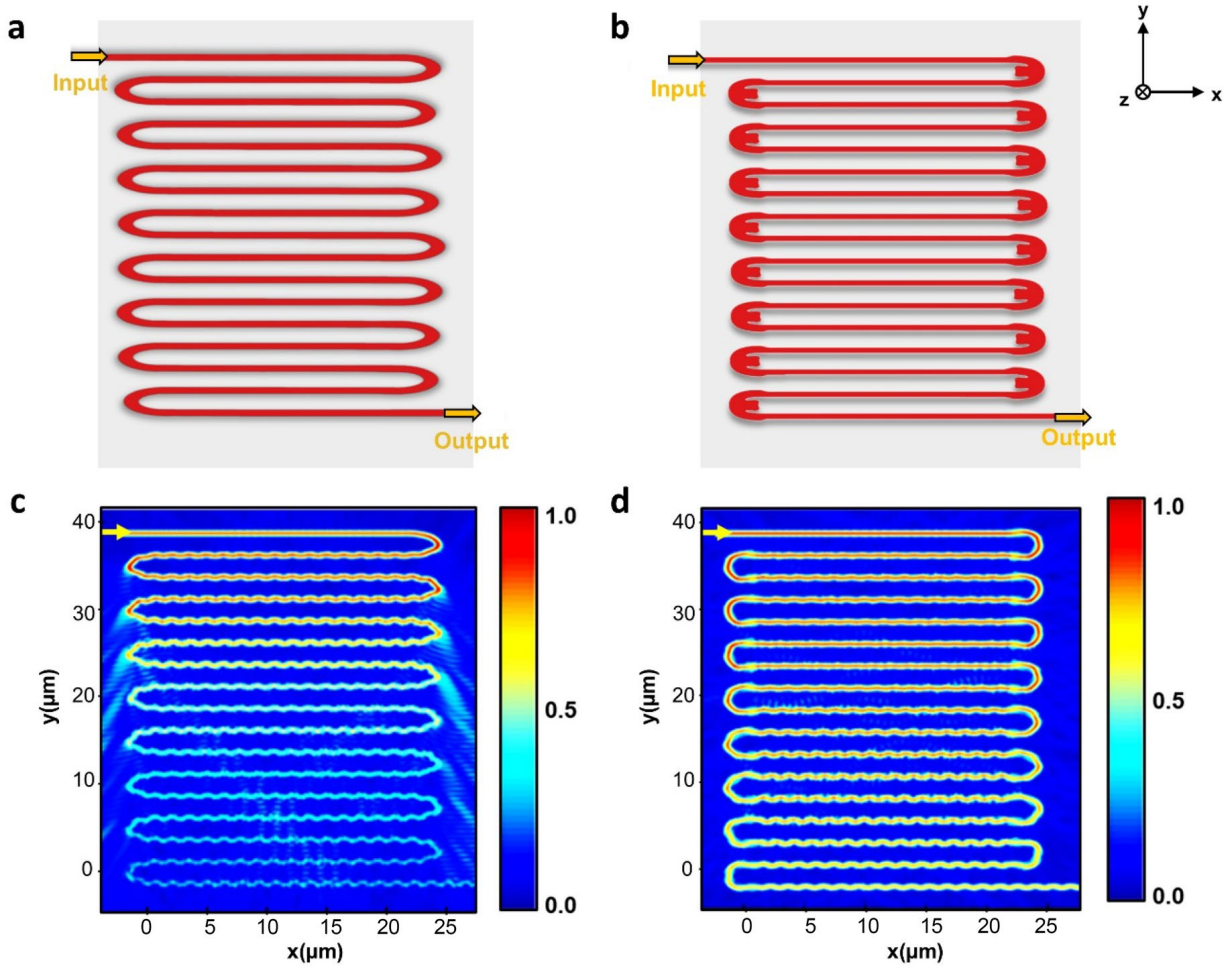
Structures of (**a**) meander line having normal U-bends with radii of 2.5 µm, and (**b**) meander line having optimized U-bends with radii of 2.5 µm. Simulated electric field profile of (**c**) meander line having normal U-bends with radii of 2.5 µm, and (**b**) meander line having optimized U-bends with radii of 2.5 µm.

To assess the effectiveness of our topology-optimized bends, we specifically focused on the U-bend 1 designs, featuring radii of 2.5 µm. Initial experiments were conducted on individual unit bends, which were then integrated into a meander line device. This meander line device consists of 16 topology-optimized U-bends connecting strip waveguides to test its scalability. Here we included a comparison of the meander line, contrasting the optimized bends with unoptimized ones of the same radius. The structural differences between the optimized and standard U-bends in the meander lines are clearly illustrated in Fig. 6a and b. In addition, the simulated electric field profiles for both meander line structures were simulated using 2.5D FDTD, presented in Fig. 6c and d. These results revealed that the meander line structure using an optimized U-bend showed considerably high transmission and reduced radiative losses, compared to the meander line employing a normal U-bend.

Leveraging the optimized designs, we fabricated the U-bend 1 device and the corresponding meander line structure on the 220 nm silicon-on-insulator (SOI) platform. This fabrication was executed through the facilities provided by the Applied Nanotools (ANT) foundry. The devices are fabricated with a single electron beam photolithography etching process with a minimum feature size of 60 nm.

**Figure 7:**
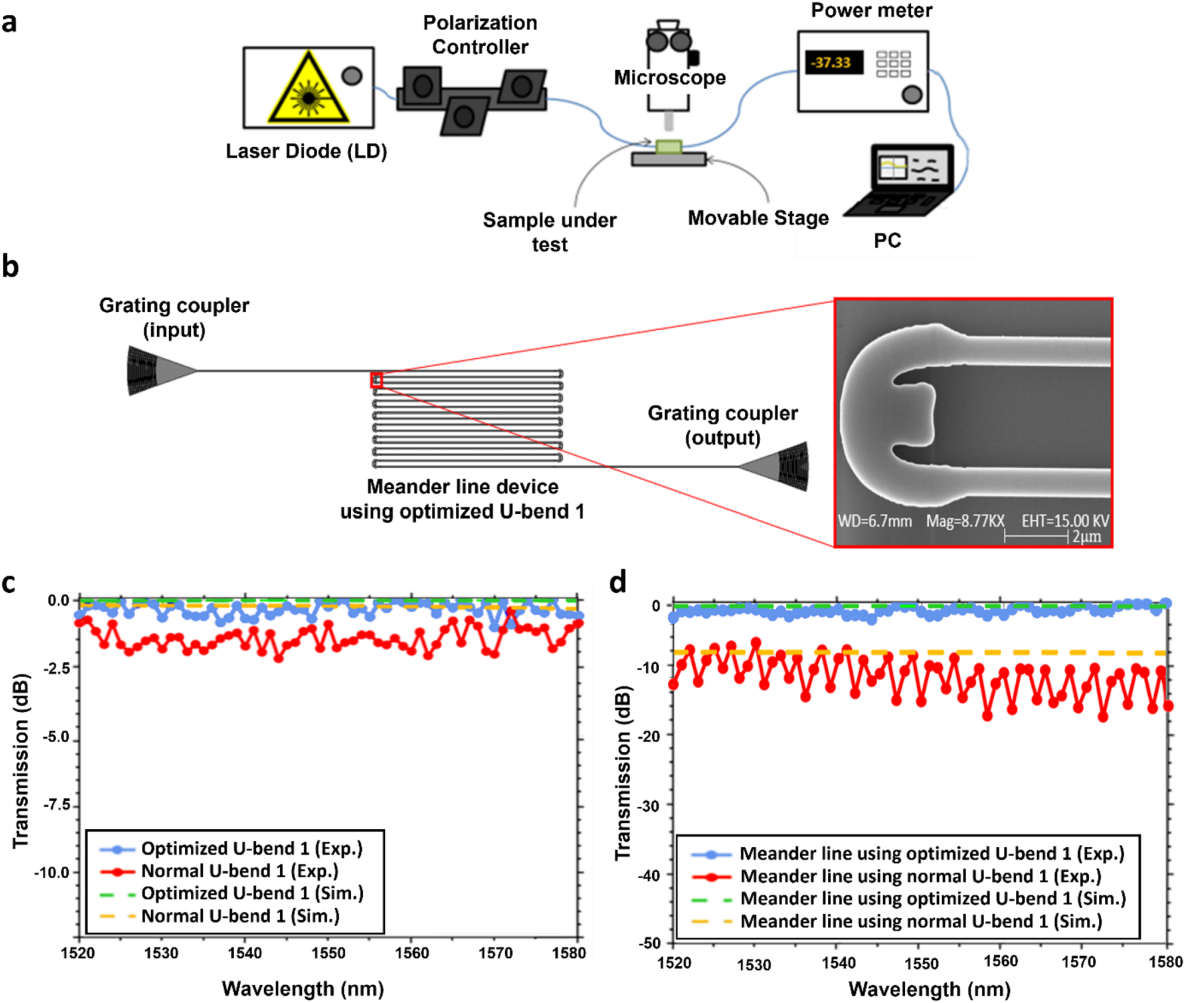
(**a**) Experimental setup for optical transmission measurement. (**b**) Layout for optimized bends-based meander line structure with SEM image of U-bend 1. (**c**) Simulated and experimental results for the transmission of optimized U-bend 1 and un-optimized U-bend 1. (**d**) Simulated and experimental results for the transmission of optimized U-bends-based meander lines and un-optimized U-bends-based meander lines.

Figure 7a depicts the experimental setup to evaluate the performance of the fabricated devices. The laser diode (LD) used has a support range from 1520 to 1580 nm. The light from the LD is routed through the optical fiber to the input grating coupler of the device. A polarization controller is used to change the polarization of light for maximum coupling with the device. A microscope is used to view the device on a chip and to couple the light from the optical fiber with the grating couplers. The output light from the output grating coupler is subsequently coupled to another optical fiber on the output side and then directed to a power meter. The power meter shows the output power level at a certain wavelength which is interfaced with LabVIEW to measure the transmission graph over a wavelength range. The GDS layout for the optimized bends-based meander lines along with the scanning electron microscope (SEM) image for the fabricated U-bend 1 obtained from ANT foundry is shown in Fig. 7b.

Figure 7c shows the transmission result of U-bend 1 in both the un-optimized and optimized forms. The average transmission of optimized U-bend 1 always stays above the un-optimized bend for all wavelength points. Specifically, the measured insertion loss of optimized U-bend 1 is 0.1 dB near the 1550 nm, whereas the un-optimized counterpart has 1.1 dB. The simulated result shows an insertion loss of only 0.05 dB for optimized U-bend 1 and 0.23 dB for un-optimized U-bend 1 at 1550 nm. Although there is a discrepancy between the obtained experimental and simulated results, the data show a comparatively better performance of inverse designed bend. Figure 7d specifies the simulated and experimental transmission spectrum of the meander line with inverse-designed bends. To verify the precise performance with the increased number of bending, a meander line structure employing 16 bends of the same radius which are not inversely designed was also fabricated and measured for its transmission response. The loss of the device consists of loss from grating couplers and waveguides therefore, a test pattern consisting of input and output grating coupler and waveguide of the same length as that of the device’s length was measured for its transmission. The loss of bend was then extracted by subtracting the loss of this test pattern from the device. The loss of bend was then extracted by subtracting the loss of this test pattern from the device. There is a high variation in experimental data points for un-optimized bends which can be attributed to scattering loss. Bends that are not carefully designed may result in mode mismatch, where the guided mode in the straight section of the waveguide does not smoothly transition into the bend. This mismatch can cause wavelength dependent scattering as the light attempts to travel the curvature. The optimized meander lines give an average experimental insertion loss of 1.23 dB, whereas the un-optimized meander lines have an insertion loss of 11.12 dB in the wavelength range from 1520 to 1580 nm. Therefore, a high increase in transmission from the inverse-designed bends is obtained and it indicates the unit optimized bend has an averaged insertion loss of 0.077 dB. The experimental data is also compared with simulated data obtained from 2.5 FDTD simulation. The simulated result shows an insertion loss of 1.23 dB for the optimized meander line, and 10.17 dB for the un-optimized meander line in the wavelength range from 1450 to 1650 nm. The comparison suggests that the experimental data lies in close vicinity of simulated data and insertion loss obtained experimentally. In alignment with recent advancements in low-loss silicon nitride PICs^34^, optimized fabrication processes could be able to mitigate the propagation losses, especially in regions involving intricate bends designs with topology optimization.

## Discussion

The performance of our inverse design bends is benchmarked by comparison with bends made with analytical methods^21,22,24^, and inverse design techniques^16^ in the literature. While it's true that our designed bends exhibit slightly higher insertion loss than certain counterparts in the literature, it's essential to note the critical distinction lying in the footprint of these structures. Some prior studies have achieved lower insertion loss, but they did so at the cost of a larger footprint, as evident in Table 1. Our bends, on the other hand, stand out for their remarkable compactness and sub-wavelength bending radius, setting a new standard for miniaturization in the realm of optical bends. It is also worth mentioning the challenges associated with the fabrication of bends made using quadratic reflectors and meta-materials methods^19,21^. The intricacies of these designs pose substantial hurdles during the fabrication process. By opting for a platform that aligns with mainstream CMOS fabrication, our designs can be feasibly produced and integrated into existing technologies. It should be noted that approaches involving a general deep learning model^35^ and differentiable morphological transforms^36^ can be further used to correct bends design and making them robust to over/under-etching thereby ensuring compliance with manufacturing constraints.

**Table 1:** Comparison of our designed bends with simulated results of bends made with different analytical approaches and inverse design methods.

Bending type	Design	Waveguide dimension (nm^2^)	Bandwidth (nm)	Device footprint (µm^2^)	Bending loss (dB)
L-bend (90°)	Bend with elliptical reflector (polymer)^37^	2300 × 2300	800–900	20 × 20	< 0.33 (TE, exp.) < 0.33 (TM, exp.)
	Optical bends^6^	400 × 220	N/A	5.0 × 5.0	< 0.002 (sim.)
	Sharp adiabatic bends^23^	2400 × 220	1530–1570	2.6 × 2.6	< 1 (exp.)
	Transformation optics-based multi-mode bends^28^	4000 × 500	N/A	79 × 79	< 0.24 (exp.)
	This work	500 × 220	1450–1650	2.5 × 2.5	< 0.06 (sim.)
				1.5 × 1.5	< 0.21 (sim.)
				1.0 × 1.0	< 3.16 (sim.)
U-bend (180°)	Bend with parabolic reflector (polymer)^37^	2300 × 2300	800–900	20 × 40	< 0.31 (TE, exp.) < 0.35 (TM, exp.)
	Optical bends^6^	400 × 220	N/A	5.0 × 10.0	< 0.013 (sim.)
	Meta-material waveguide bends^38^	500 × 220	1450–1650	3.0 × 3.0	< 1.87 (TE, exp.) < 1.37 (TM, exp.)
	This work	500 × 220	1450–1650	3.0 × 3.6	< 0.07 (sim.)
				2.5 × 2.5	< 0.60 (sim.)
				1.5 × 1.5	< 0.78 (sim.)
			1520–1580	3.0 × 3.6	< 0.077 (exp.)

While our analysis is carried out for waveguide with a width of 500 nm, it can be easily extended to other commonly used waveguide widths for a better comparison with the other works in literature. As we explore different waveguide widths, we anticipate variations in the topologically optimized structures. The interplay between the waveguide width and the optimal topology arises from the complex interactions of electromagnetic fields within the waveguide. Different widths may exhibit unique electromagnetic behaviors, influencing the evolution of optimized structures to ensure efficient light propagation. It is expected to obtain similar performances in terms of suppressing waveguide bending losses if one employs inverse design method for waveguides with a different width.

Furthermore, it's important to note that this topology optimization approach can be employed not just for TE mode, as demonstrated in our study, but also for optimizing bending structures that support Transverse Magnetic (TM) mode. When we conducted 2D simulations specifically for TM mode, we observed distinct differences in the shapes of bending structures compared to those supporting TE mode. This observation implies that our approach is exceptionally versatile and applicable across different modes, potentially generating structures that we may not have predicted.

The significance of our study becomes even more apparent when considering the challenges associated with sharp bending in optical waveguides. Efficiently routing light through sharp bends is a complex endeavor, and topology optimization proved effective for this challenge. One might question the purpose of bends with an extremely small footprint, as such designs lead to high insertion losses even after optimization. However, to attain a lower limit for footprint and satiate our scientific curiosity about how the optimization performs in a very constrained space, we made these designs. When compared to an un-optimized silicon bend of the same radius, our designed bends exhibit a tremendous improvement, showcasing the efficacy of our approach in enhancing signal transmission through these highly compact structures.

Moreover, our measurement results offer promising prospects for practical applications. The incorporation of meander lines with optimized bends not only reduces the footprint of delay lines significantly but also ensures robust signal transmission over extended distances. While there is a marginal increase in measured insertion loss compared to the simulated data, it's crucial to attribute this to the inherent challenges of real-world measurements. Factors such as misalignment of fiber connectors, imperfections in the coupling between devices and fibers, or minor inaccuracies in measurement instruments can contribute to these discrepancies.

## Conclusion

Our study introduces a novel approach to the design of waveguide L-bends and U-bends through inverse design topology optimization. In the rapidly evolving landscape of photonic chip technology, where applications like on-chip optical computing, programmable photonics, and photonic neural networks are becoming prominent, efficient light routing within these chips is paramount. Two critical challenges in this context are reducing the bending radii of waveguide bends to enhance integration density while minimizing bending losses and ensuring these bends operate across a broad wavelength range. Our study addresses these challenges specifically in the realm of silicon photonics, which is highly compatible with the mature CMOS platform.

To achieve this, we employed topology optimization, leveraging its advantage in utilizing design space and time efficiently. Our optimization process focused on ultra-compact waveguide bends, ranging from 3 µm × 3.6 µm down to 1 µm × 1 µm. The designed bends not only demonstrated a reduction in insertion loss but also preserved mode characteristics compared to un-optimized conventional bends of the same radii. The significant achievement of our study was an average 50% decrease in maximum insertion loss when compared to un-optimized bends with similar footprints. Furthermore, through fabrication and experimental testing, we highlighted the superiority of meander line structures incorporating optimized U-bends over those made with un-optimized bends of the same radius. This superiority was evident in the optimized meander line's substantially lower insertion loss and reduced radiated power at each U-bend. In comparison to existing research, our work stands out for its dual focus on reducing insertion loss and operating across a wide wavelength range while working in a small design footprint. Additionally, our study contributes valuable insights into the practical implementation of meander line structures, showcasing the benefits of optimized bends in real-world applications.

## Data Availability

The datasets generated and/or analyzed during the current study are available from the corresponding author upon reasonable request.
